# Discovery of Small Molecules as Membrane-Bound Catechol-*O*-methyltransferase Inhibitors with Interest in Parkinson’s Disease: Pharmacophore Modeling, Molecular Docking and In Vitro Experimental Validation Studies

**DOI:** 10.3390/ph15010051

**Published:** 2021-12-31

**Authors:** Pedro Cruz-Vicente, Ana M. Gonçalves, Octávio Ferreira, João A. Queiroz, Samuel Silvestre, Luís A. Passarinha, Eugenia Gallardo

**Affiliations:** 1 CICS-UBI, Health Sciences Research Centre, Universidade da Beira Interior, 6201-001 Covilha, Portugal; pedromvcruz@hotmail.com (P.C.-V.); ggmargarida@gmail.com (A.M.G.); octavioferreira.2@gmail.com (O.F.); jqueiroz@ubi.pt (J.A.Q.); 2 UCIBIO—Applied Molecular Biosciences Unit, Departamento de Química, Faculdade de Ciências e Tecnologia, Universidade NOVA de Lisboa, 2829-516 Caparica, Portugal; 3 Associate Laboratory i4HB—Institute for Health and Bioeconomy, NOVA School of Science and Technology, Universidade NOVA de Lisboa, 2819-516 Caparica, Portugal; 4 CNC—Center for Neuroscience and Cell Biology, University of Coimbra, 3004-504 Coimbra, Portugal; 5 Laboratório de Fármaco-Toxicologia—UBIMedical, Universidade da Beira Interior, 6200-001 Covilha, Portugal

**Keywords:** Parkinson’s disease, catechol-*O*-methyltransferase, inhibitors, bioinformatics, pharmacophore modeling, molecular docking, cytotoxicity

## Abstract

A pharmacophore-based virtual screening methodology was used to discover new catechol-*O*-methyltransferase (COMT) inhibitors with interest in Parkinson’s disease therapy. To do so, pharmacophore models were constructed using the structure of known inhibitors and then they were used in a screening in the ZINCPharmer database to discover hit molecules with the desired structural moieties and drug-likeness properties. Following this, the 50 best ranked molecules were submitted to molecular docking to better understand their atomic interactions and binding poses with the COMT (PDB#6I3C) active site. Additionally, the hits’ ADMET properties were also studied to improve the obtained results and to select the most promising compounds to advance for in-vitro studies. Then, the 10 compounds selected were purchased and studied regarding their in-vitro inhibitory potency on human recombinant membrane-bound COMT (MBCOMT), as well as their cytotoxicity in rat dopaminergic cells (N27) and human dermal fibroblasts (NHDF). Of these, the compound ZIN27985035 displayed the best results: For MBCOMT inhibition an IC_50_ of 17.6 nM was determined, and low cytotoxicity was observed in both cell lines (61.26 and 40.32 μM, respectively). Therefore, the promising results obtained, combined with the structure similarity with commercial COMT inhibitors, can allow for the future development of a potential new Parkinson’s disease drug candidate with improved properties.

## 1. Introduction

With the increasing life expectancy of the population, neurodegenerative disorders (ND) such as Alzheimer’s and Parkinson’s disease (PD), are becoming more common and recognized as a social problem to modern societies [1]. PD is characterized by a progressive neurodegeneration in the central nervous system (CNS) that involves a loss of dopaminergic neurons in the brain and nowadays is considered one of the main causes of disability and mortality [2]. The current drug therapy mostly targets symptomatic relief, mainly aiming to restore dopaminergic function [2]. However, this strategy is not capable of stopping the progression of the disease and the advance of neurodegeneration and symptom aggravation [2]. To date, the most effective drug combination used in PD treatment is levodopa (*L*-DOPA) combined with aromatic *L*-amino acid decarboxylase and catechol-*O*-methyltransferase (COMT) inhibitors to restore dopaminergic brain levels [2]. However, their long-term administration is generally associated with harmful side effects that affect the patient’s quality of life, and a loss in pharmacological effect is usually observed over time [3]. Therefore, there is an increasing demand to develop novel PD drug candidates. Considering the active role that COMT holds in *L*-DOPA metabolism, both in the periphery and in CNS, converting more than 90% of administered *L*-DOPA before reaching the brain, the discovery of a novel COMT inhibitor with low toxicity, high inhibitory potency, and selectivity towards the CNS is of high interest. Specifically, COMT is a magnesium-dependent enzyme that mediates the removal of a methyl group from *S*-adenosyl-_L_-methionine (SAM) to a catecholic substrate, affording *O*-methylated products and *S*-adenosylhomocysteine (AdoHcy) [4]. Physiologically, this enzyme appears as two distinct isoforms, a soluble isoform (SCOMT) mainly expressed in the peripheric systems, such as the liver, kidney, and intestines, and a membrane-bound (MBCOMT) isoform with a major prevalence in the central nervous system (CNS) [5,6,7]. Despite the high similarity shared by both isoforms, SCOMT has a higher enzymatic activity capacity, whereas MBCOMT has a higher substrate affinity (Km), especially towards catecholamines like dopamine and epinephrine [2,5]. Since the discovery of the pharmacological benefit of COMT inhibition, several families of molecules have been reported in the literature. The first generation was composed of molecules with a high structure similarity with known COMT substrates, bearing a catechol moiety [8,9]. However, despite some promising in-vitro results, in in-vivo testing, the compounds displayed a high toxicity and low inhibitory potency [8]. So, a new group of inhibitors was proposed, mainly formed by di-substituted catechols, where entacapone and tolcapone, commercial COMT inhibitors used in a clinical setting at the moment, were included [9]. These compounds showed enhanced potency, generally in the nanomolar range, especially caused by the presence of electron-withdrawing groups at the *ortho* position to a hydroxyl group of the catechol moiety [8]. Despite its clinical use and pharmacological properties, including the capacity to cross the blood–brain barrier (BBB) and thus inhibit central COMT, tolcapone is also associated with high hepatoxicity, limiting its use in more advanced cases of PD progression. In contrast, entacapone, a strictly peripheric inhibitor, is less potent, but has less toxicity and was considered as the first line of treatment for many years [10]. Recently, another peripheric COMT inhibitor, opicapone, was approved for clinical use. This drug displays a higher bioavailability than other commercial inhibitors and leads to stable and sustained *L*-DOPA plasma levels for over 24 h periods, which allows for a big improvement in the life quality of PD patients [11]. Regardless of the intense research to improve levodopa bioavailability, only a couple of novel adjunct compounds acting through COMT inhibition have been approved in the last few decades [3]. So, it is imperative to find new COMT inhibitors with the ability to cross the BBB and promote a synergic effect (peripheral/central) with a high safety profile. Considering the need to combine new technologies to support the design of novel drugs, bioinformatics is gaining outstanding relevance in almost all therapeutical areas. It is no different for COMT inhibition, and since the 1990s many researchers have employed some of these tools to discover and/or aid in the design of new molecules. In this context, Vidgren et al. were one of the first research groups to employ the use of computational tools to study the molecular interactions of potential inhibitors with the COMT catalytic site [12]. The acidity of both catechol hydroxyl groups and the lipophilicity of the inhibitors side chains were demonstrated to play an important role in the molecules’ binding affinity, later confirmed by the determination of the first COMT crystal structure [13]. Lautala et al. [14], by comparing unsubstituted catechol and pyrogallol, first-generation inhibitors, and their substituted derivatives, demonstrated that the turnover rate modification of the molecules was inversely proportional to their binding affinity to the protein active site. Moreover, several research groups studied the inhibitory potential of flavonoids through molecular docking [15], dynamic simulations [16], and QSAR (quantitative structure–activity relationship) studies [17]. Similar strategies were employed for the “second generation” inhibitors, as reported by Palma et al., who applied molecular docking to understand the molecular interactions of nebicapone [18], a nitrocatechol inhibitor, and the *O*-regioselectivity of the enzyme for BIA 3-228 and BIA 8-176, two other nitrocatecholic-type inhibitors [19]. Another class of inhibitors that was studied was the bisubstrate inhibitors, molecules that target simultaneously both the SAM and the substrate binding sites [20]. In fact, both Lerner et al. [21] and Paulini et al. [22] used molecular docking to evaluate the effect of several substituents on the binding affinity with COMT. Moreover, Lee et al., through homology modeling of the 3D structure of rat COMT, developed several bisubstrate inhibitors performed molecular docking to analyze putative binding affinities [23]. Furthermore, Ellerman et al. used structure-based drug design (SBDD) methodologies to evaluate the binding affinity of multiple compounds with the COMT 3D structure [24,25]. Recently, garcinol, a natural product with an in-vitro effect identical to the one observed with tolcapone, was also studied in silico and it was shown that it can form identical atomic interactions with COMT when compared to tolcapone [26]. A slightly different approach was used by Jatana et al. by employing pharmacophore modeling and ligand screening analysis to obtain molecules with a high potential to interact with COMT [27]. All studies were performed with the soluble isoform of mouse or human COMT. So, due to the difficulty of obtaining the 3D structure of membrane proteins, bioinformatics approaches have been extensively used to explore the conformational space of a ligand in the binding pocket of the selected target protein. At present, more than 100 3D SCOMT crystal structures can be found in the Protein Data Bank (https://www.rcsb.org/, accessed on 27 November 2021), and they provided essential information about the atomic interactions formed between the protein active site and substrates/inhibitors. Nevertheless, the MBCOMT structure has not been solved to date and no structures between SCOMT and MBCOMT coupled to new molecules have been reported in the literature. So, in this work, the resolution of the crystal structure of the SCOMT variant [28] was the base for structure-based virtual screening and pharmacophore modeling studies to find potential COMT inhibitors with increased selectivity towards MBCOMT. This study, using a combination of structure- and ligand-based drug design approaches to select new promising candidates to be studied in-vitro for both MBCOMT inhibition and cell line cytotoxicity, aims to discover novel PD drug candidates with interest for further studies.

## 2. Results

### 2.1. Pharmacophore Modeling

**Figure 1:**
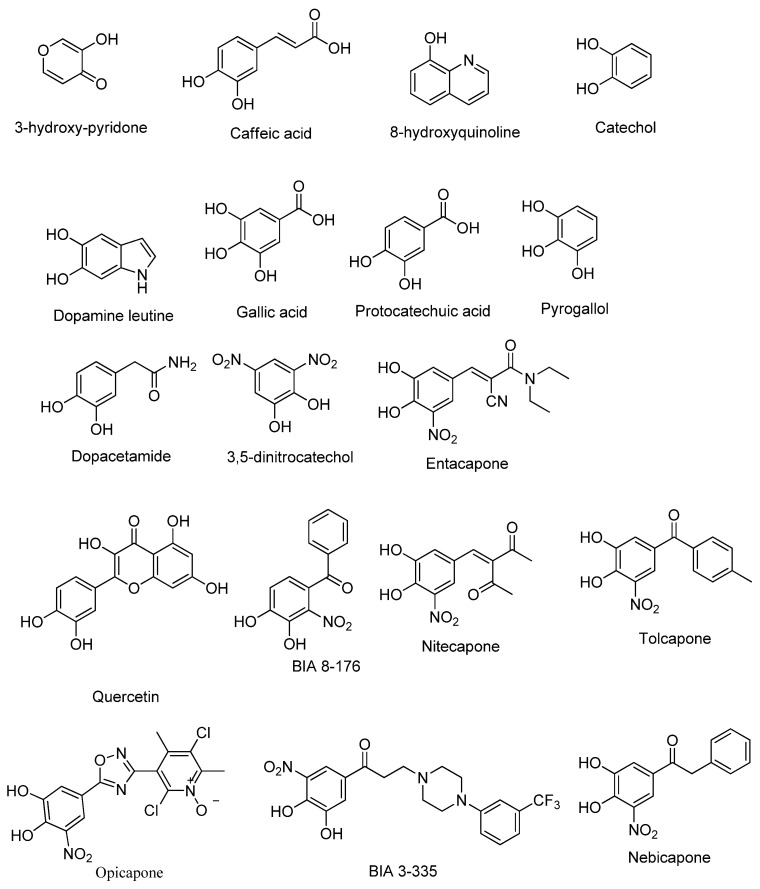
2D structures of the selected COMT inhibitor training set.

**Figure 2:**
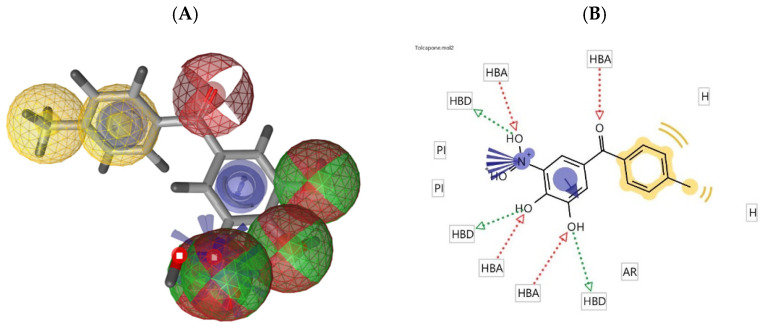
(**A**) Pharmacophore model of tolcapone generated by LigandScout (hydrogen bond donor: green sphere, hydrogen bond acceptor: red sphere, ionizable area: blue asterisk, and aromatic rings: yellow); (**B**) 2D representation of the pharmacophore features of tolcapone.

Employing a ligand-based molecular modeling strategy, a pharmacophore containing the main structural moieties of the training set (Figure 1) was generated to find new potential COMT inhibitor drug candidates. By merging the essential features of the selected molecules (Figure 1), the model explored the number of hydrogen bond donors/acceptors as well as the aromatic and hydrophobic groups involved in the interaction with the COMT active site, generating a model very similar to the interactions performed by the commercial COMT inhibitor tolcapone, as shown in Figure 2 [29]. 

### 2.2. Database Searching

From the 10 best scored hypotheses, only the best scored model was selected to advance for further studies. The selected pharmacophore was loaded into the ZINCPharmer software and a virtual screening of the ZINC purchasable database was performed, selecting the 100 best scored hit molecules. These 100 compounds were selected considering literature data, with a focus on the knowledge of the main molecules with COMT inhibitory effects (e.g., compounds presented in Figure 1). From an initial set of 21,777,093 compounds, the compounds with RMSD (root-mean-square deviation) ranging from 0.5 to 1.5 Å were the best ranked compounds, and commercial COMT inhibitors like tolcapone and opicapone were also considered hit molecules, improving the reliability of the obtained data. In Supplementary Material Table S1 the RMSDs, molecular weight, and routable bonds of some of the hit molecules selected are displayed.

### 2.3. Molecular Docking Studies

**Figure 3:**
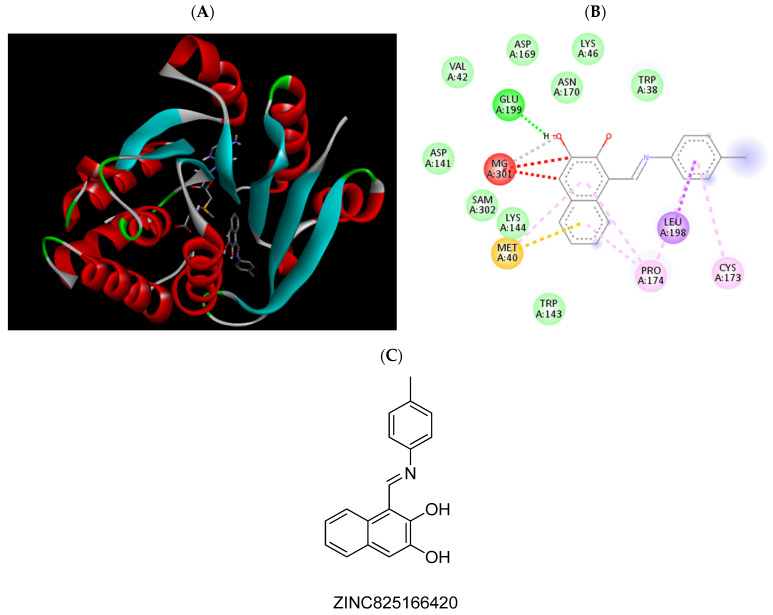
(**A**) Binding modes of ZINC825166420 with the crystal structure of human COMT complexed with SAM and Mg^2+^, (**B**) receptor–ligand interactions, (**C**) 2D representation of ZINC825166420.

**Figure 4:**
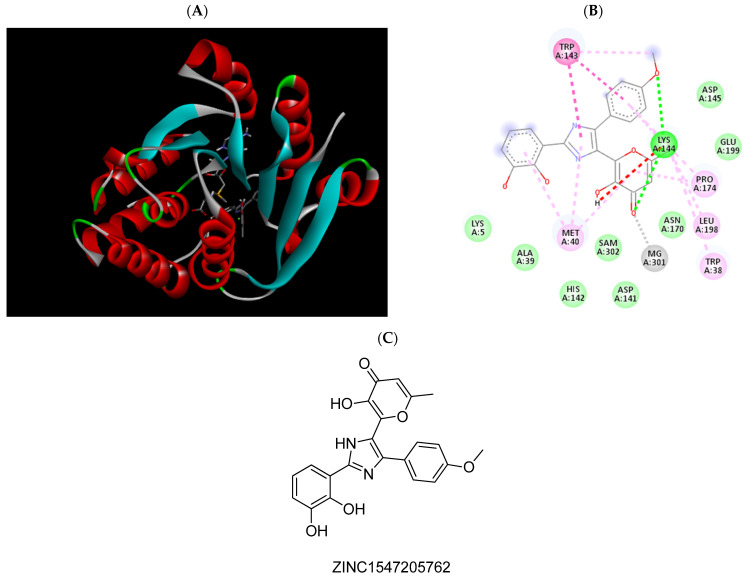
(**A**) Binding modes of ZINC1547205762 with the crystal structure of human COMT complexed with SAM and Mg^2+^, (**B**) receptor–ligand interactions, (**C**) 2D representation of ZINC1547205762.

**Figure 5:**
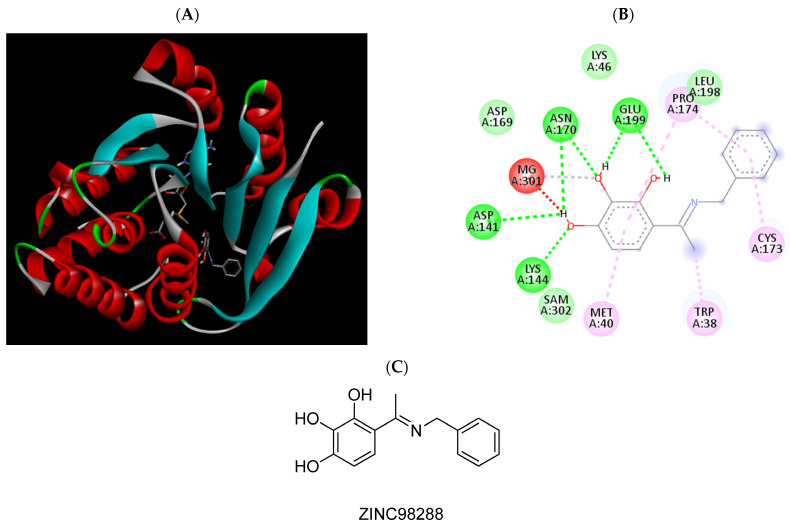
(**A**) Binding modes of ZINC98288 with the crystal structure of human COMT complexed with SAM and Mg^2+^, (**B**) receptor–ligand interactions, (**C**) 2D representation of ZINC98288.

**Figure 6:**
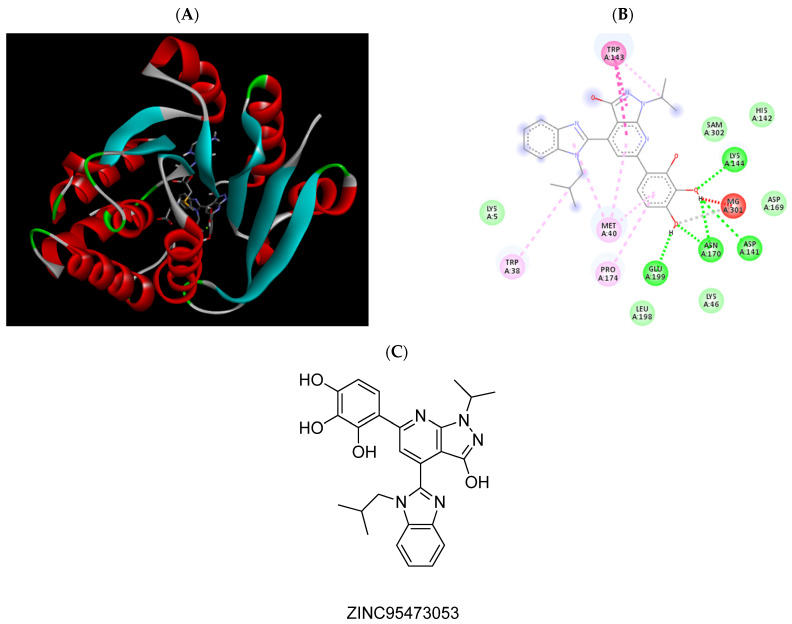
(**A**) Binding modes of ZINC95473053 with the crystal structure of human COMT complexed with SAM and Mg^2+^, (**B**) receptor–ligand interactions, (**C**) 2D representation of ZINC95473053.

**Figure 7:**
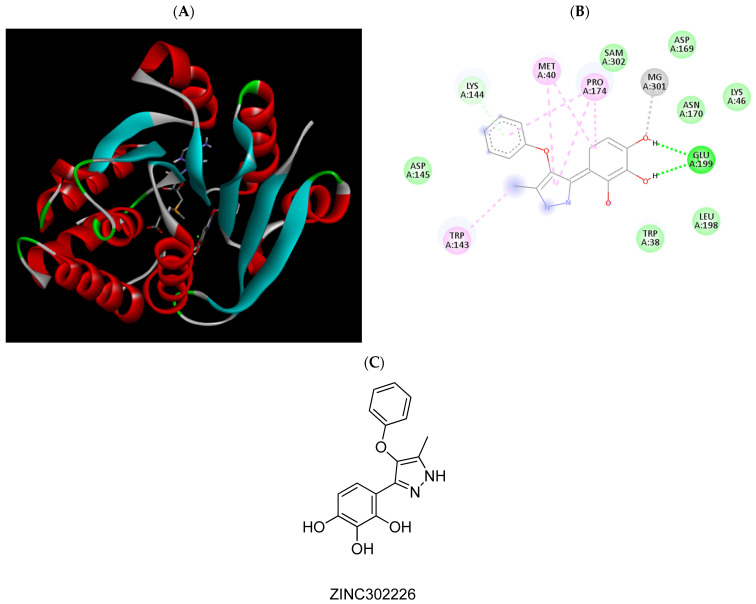
(**A**) Binding modes of ZINC302226 with the crystal structure of human COMT complexed with SAM and Mg^2+^, (**B**) receptor–ligand interactions, (**C**) 2D representation of ZINC302226.

**Figure 8:**
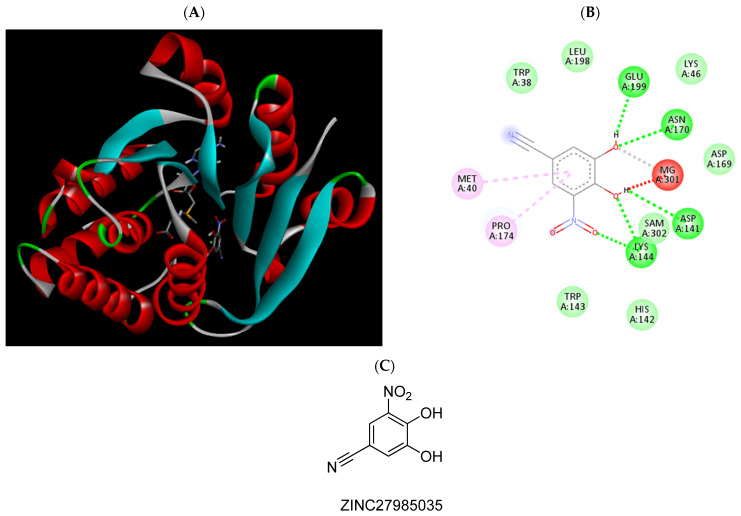
(**A**) Binding modes of ZINC27985035 with the crystal structure of human COMT complexed with SAM and Mg^2+^, (**B**) receptor–ligand interactions, (**C**) 2D representation of ZINC27985035.

**Figure 9:**
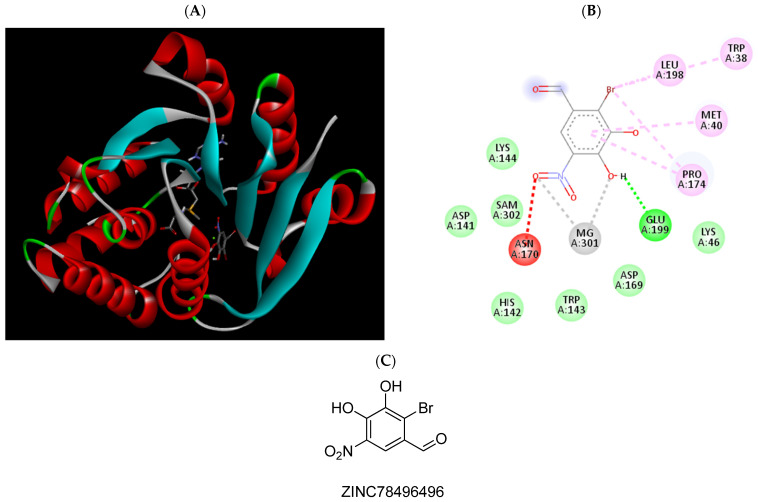
(**A**) Binding modes of ZINC78496496 with the crystal structure of human COMT complexed with SAM and Mg^2+^, (**B**) receptor–ligand interactions, (**C**) 2D representation of ZINC78496496.

**Figure 10:**
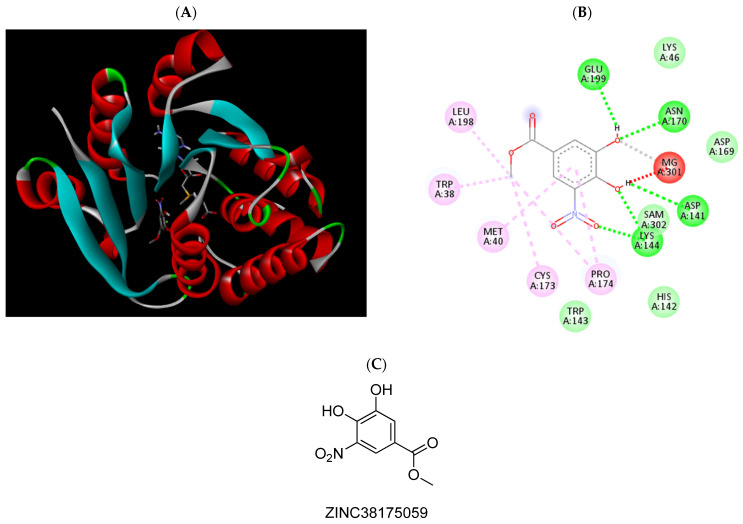
(**A**) Binding modes of ZINC38175059 with the crystal structure of human COMT complexed with SAM and Mg^2+^, (**B**) receptor–ligand interactions, (**C**) 2D representation of ZINC38175059.

**Figure 11:**
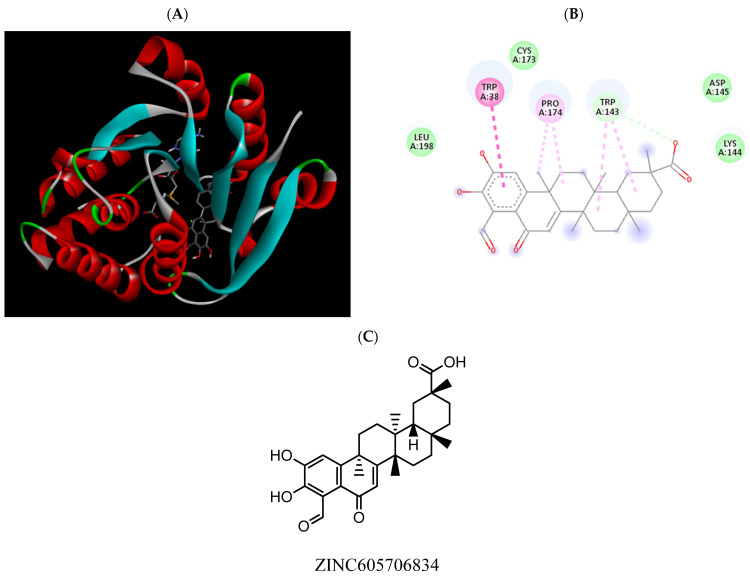
(**A**) Binding modes of ZINC605706834 with the crystal structure of human COMT complexed with SAM and Mg^2+^, (**B**) receptor–ligand interactions, (**C**) 2D representation of ZINC605706834.

**Figure 12:**
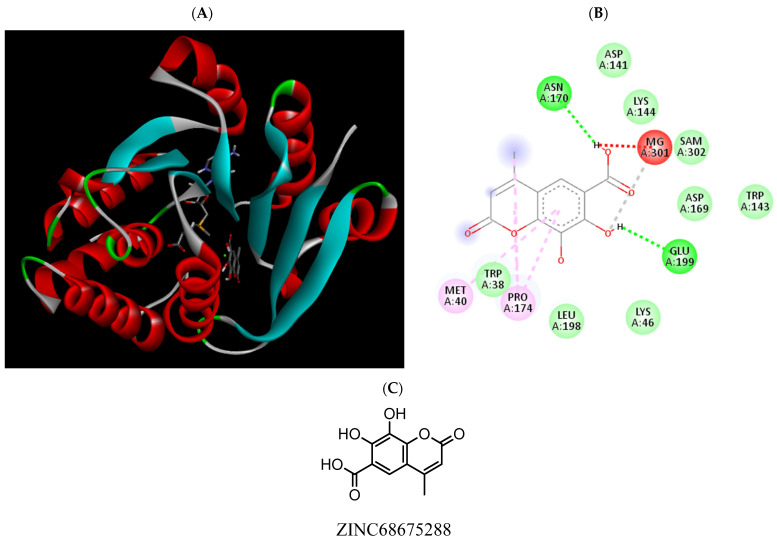
(**A**) Binding modes of ZINC68675288 with the crystal structure of human COMT complexed with SAM and Mg^2+^, (**B**) receptor–ligand interactions, (**C**) 2D representation of ZINC68675288.

Molecular docking was used to better comprehend and validate the provisional results obtained in Section 2.2, filtering potential false negatives and studying in detail the atomic interactions formed with the COMT active site. To do so, the 3D structure of COMT (PDB#6I3C) co-crystallized with the cofactors SAM and Mg^2+^ ions and the inhibitor 3,5-DNC, identified as the reference compound, was used [28]. To date, this structure has the best resolution of all the structures of COMT deposited in PDB (data checked on 27 November 2021). Structurally, the COMT catalytic site is surrounded by the “gatekeeper” residues Trp143 and Pro174, which ensure the correct orientation of the substrate, the Mg^2+^ ions, and the SAM cofactors, as well as residues Lys144 and Glu199, which are involved in substrate binding [30]. The methodology used was validated through a re-docking of the co-crystallized reference COMT inhibitor 3,5-DNC into the protein active site through AutoDock Tools software, obtaining an RMSD value of 1.6 Å and a binding energy of −4.85 kcal/mol. These results indicate that the procedure used was able to reproduce the crystallographic complexes in a very precise approach. The hit molecules were also subjected to the same procedure and were ranked based on their molecular interactions formed with the COMT active site by both hydrophobic and electrostatic interactions, as summarized in Supplementary Material Table S2. In Table 1 the atomic interactions of some of the most promising scoring molecules are displayed in more detail, as well as in Figure 3, Figure 4, Figure 5, Figure 6, Figure 7, Figure 8, Figure 9, Figure 10, Figure 11 and Figure 12. A special focus was brought to the molecules that could form interactions with the most important amino acids of the protein active site, mainly with Lys144 and Glu199 by hydrogen bonding and with Trp38, Trp143, and Trp174 by hydrophobic interactions. Additionally, the binding energy values were also evaluated, namely, in comparison with the reference compound 3,5-DNC.

### 2.4. ADMET Property Prediction

The ADMET properties were predicted for the 50 best scored compounds in the virtual screening. Table 2 summarizes the results for the 10 most relevant selected molecules in several categories, such as absorption, distribution, metabolism, excretion, and predicted toxicity, obtained in the webserver pkCSM. These included the BBB (blood–brain barrier) and CNS (central nervous system) permeability, which are considered important attributes of a PD drug, where most of the tested compounds were in the lower limit of being considered a permeable drug. The human intestinal potential absorption is indicative of higher intestinal absorption, which is a factor to take into account in cases of oral drug administration (the results were in most cases percentages above 80%, a very positive result). Regarding the PgP (P-glycoprotein) interaction, a protein involved in cells’ internal efflux mechanisms for xenobiotic substances, the compound properties both as a substrate and/or inhibitor were assessed, yielding positive data due to low indications of an interaction with these proteins. The inhibition of several cytochrome P450 enzymes was also predicted, specifically for the CYP2C9, CYP2D6, CYP3A4, CYP1A2, and CYP2C19 variants, where most compounds did not inhibit any of these enzymes, with the exception of CYP2C19, where almost half of the studied compounds inhibited this isoenzyme. For AMES toxicity, which states the mutagenicity of a compound evaluated, we concluded that only a few compounds were predicted as being potentially mutagenic. The compounds LD_50_ (median lethal dose) and LOAEL (lowest observed adverse effect level) were predicted as being similar to other clinically used drugs, such as some of the commercial COMT inhibitors. Finally, hepatotoxicity was also studied, with only one negative result observed, for the compound ZINC825166420. These results, despite being in-silico data, are very interesting, with some very positive results that can potentially indicate some of the in-vitro and in-vivo results. However, they can only be validated with further testing.

After this analysis, based on binding energies and atomic interactions formed with the COMT active site (Section 2.3), commercial availability, and the structure similarity with commercial or previously reported COMT inhibitors, 10 compounds were selected to be tested further.

### 2.5. In-Vitro MBCOMT Assays

**Figure 13:**
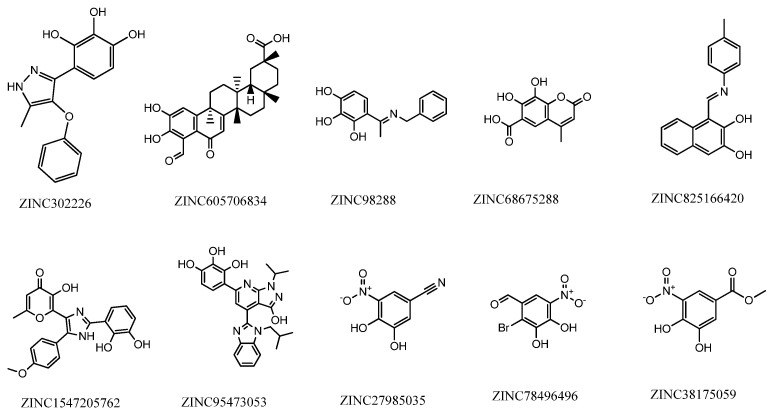
Structure and identification of the studied compounds.

Despite structural-based drug design tools being able to predict the binding modes and affinity of new molecules to the targets with significant accuracy and efficiency [31], an in-vitro validation of the best modeling results must be performed. Therefore, according to the best hits given by the computational models, 10 compounds were chosen to perform the enzymatic inhibition assay (Supplementary Material Table S3). As previously mentioned, our group purchased the most promising compounds with purity above 99.5% according to the manufacturer. The compounds used in this study are presented in Figure 13, with the respective identification. 

From these previous studies, a first screening of MBCOMT inhibitory effects allowed us to select the compounds with the five best inhibitory profiles for concentration-response studies to calculate IC_50_ values, which are presented in Table 3. From the analysis of the results, the lowest IC_50_ was determined for the compound ZINC27985035, followed by ZINC78496496. 

### 2.6. Cytotoxicity Studies

The neuronal cytotoxicity is an important parameter to evaluate considering the target organ of action of anti-Parkinsonian agents, namely, for potential COMT inhibitors. Therefore, the effect of the selected compounds was evaluated against dopaminergic neuronal cells (N27) by the 3-(4,5-dimethylthiazol-2-yl)-2,5-diphenyltetrazolium bromide (MTT) colorimetric assay, as previously described [35]. To do so, the cells were exposed to different concentrations of the compounds during 48 h, and after the MTT assay (Supplementary Material Figures S1 and S2), their half maximal inhibitory concentrations (IC_50_) were calculated by considering the data from concentration-response curves (Table 4). In addition, the cytotoxicity of these compounds to normal human dermal fibroblasts (NHDF) was also assessed by the same methodology to obtain further data on the in-vitro safety to non-tumoral cells existing outside of the CNS. The clinical drug 5-fluorouracil (5-FU) was included in the study for comparative purposes. 

## 3. Discussion

Aiming to discover new compounds with potential interest in PD therapy, we employed a previously described crystal structure of SCOMT [28] to develop a pharmacophore to find potential new COMT inhibitors with higher selectivity to MBCOMT. Then, we used the pharmacophore developed to computationally find molecules of interest at the ZINC purchasable database. Next, the 100 best scored compounds achieved in this database search were evaluated for their drug-likeness properties and by molecular docking followed by an analysis of relevant ADMET parameters. Considering the results of these studies as well as commercial availability, 10 compounds were selected and acquired for further in-vitro studies, taking into account the obtained binding energy and the main interaction promoted with the target amino acids (Supplementary Material Table S2). Therefore, by a combination of in-silico structure- and ligand-based drug design strategies, new hit molecules were selected. In this context, it is important to mention that structural-based drug design tools are being widely used to predict the position and affinity of new molecules to the target protein with considerable accuracy and efficiency, among other studies [31]. Nevertheless, despite extensive in-silico studies, the in-vitro validation of the best predicted modeling results must be performed. To do so, we carried out experiments to evaluate the inhibition of MBCOMT activity (Table 3) as well as the cytotoxicity (Table 4) of the compounds with higher relevancy in dopaminergic and fibroblast cells, and we observed interesting results that are discussed below.

COMT inhibitors can be divided into the “first generation,” associated with toxic properties and weak or nonselective activity, and the “second generation,” with nitrocatecholic structure, which includes the inhibitors 3,5-dinitrocatechol (3,5-DNC), opicapone, nitecapone, nebicapone, entacapone, and tolcapone [36,37]. The last two proved to be potent COMT inhibitors with similar IC_50_s of approximately 250 nM [36]. Although entacapone has been considered relatively safe, it only acts peripherally [10]. In contrast, tolcapone, the only commercial COMT inhibitor able to cross the BBB, is used with caution due to safety concerns regarding liver toxicity [38]. In this work, the MBCOMT inhibition potencies of the five selected compounds were determined based on the sigmoidal curve of dose vs. response drawn and expressed as IC_50_ values. As shown in Table 3, the majority of the tested compounds in this work presented an IC_50_ value below 1000 nM, with the compound ZINC825166420 presenting the highest IC_50_ (1538 nM), followed by ZINC302226. Although the compound ZINC302226 was computationally predicted to be the strongest inhibitor to be tested, it presented a relatively high IC_50_ value (1083 nM) compared to the reported values for entacapone or tolcapone [2,5,6]. On the other hand, the compounds ZINC98288, ZINC27985035, and ZINC78496496 had the lowest IC_50_ values (below 1 µM). In fact, the derivative ZINC27985035 presented the lowest IC_50_ value achieved (17.6 nM), similar to that determined for 3,5-DNC when the recombinant SCOMT was used. However, if we consider liver S- and MBCOMT as the protein source, the obtained value is much lower than those reported for both entacapone and tolcapone, which share structural similarities with ZINC27985035. A detailed analysis of the scientific literature also allowed for the identification of compounds with a structure similar to ZINC302226 [37] and ZINC78496496 [39] as COMT inhibitors. For the last one, the only difference was the presence a chlorine group instead of bromine. In addition, via a deep bibliographic search and analysis, we found that the compound ZINC27985035, the structure with the highest MBCOMT inhibition in our studies, has already been described as a COMT inhibitor [39,40]. Despite this, it is important to mention that the structural similarity of ZINC302226 and ZINC78496496 to previously described COMT inhibitors and that ZINC27985035 was already published as an inhibitor of this enzyme can be considered a support to our in-silico strategy applied to discovering new compounds targeting this enzyme. In addition, and more importantly, the inhibition studies performed for these three compounds were performed against the enzyme isolated from animal tissues and not for cell extracts of recombinant human MBCOMT. However, we did not find any information in the literature concerning the compounds ZINC98288 and ZINC825166420 as COMT inhibitors. Therefore, taking into account the results described in the present work and despite the inhibition levels observed in our assays and the need for more studies, especially at the in-vitro and in-vivo levels, we consider that these compounds may have potential interest in the context of PD and can be new skeletons for further exploration in the medicinal chemistry of COMT inhibitors. 

Concerning the cytotoxicity (Table 4), overall, with the exception of ZINC825166420, it can be considered that the majority of the tested compounds did not exhibit relevant cytotoxicity in both cell lines. The lowest IC_50_ values were observed for 5-FU, as expected. 

Therefore, these results can be of high interest, revealing a marked selectivity when considering the effects of the studied compounds on MBCOMT inhibition in comparison with their cytotoxicity.

Overall, considering the pros and cons of the existing commercial COMT inhibitors, with further structural optimization, pharmacokinetics, and bioavailability studies, these molecules could have potential to improve the day-to-day life of PD patients. However, more studies are needed to address this possibility.

## 4. Materials and Methods

### 4.1. Ligand Selection

From an initial set of 100 molecules with known COMT inhibitory properties, the 18 most promising compounds were selected to integrate into the study training set (Figure 1) [9]. This selection was carried out based on the compounds’ structure and existing in-silico data regarding their interaction with COMT, including molecular docking and molecular dynamics interactions with the protein active site [29] and the ADMET (absorption, distribution, metabolism, excretion, and toxicity) property prevision, as well as their in-vitro and in-vivo COMT inhibitory potency and bioavailability [9]. The 18 selected most promising compounds, used as the training set, were drawn in ChemDraw (v. 12.0), and their conformational energies were minimized using the MM2 force field in Chem3D (v. 12.0) to improve the reliability of the obtained results. Moreover, the 3D protein structure determined by X-ray diffraction of human SCOMT (PDB#6I3C) was retrieved from the Protein Data Bank (PDB) [28]. This COMT structure was determined with an atomic resolution of 1.34 Å, contained 232 amino acids in a single chain, and was co-crystallized with the cofactors Mg^2+^, SAM, and the inhibitor 3,5-DNC [28]. For this study, the computational 3D protein structure was prepared using Chimera (v. 1.13), removing the crystallographic water molecules, adding the hydrogen atoms, and minimizing the conformational energy using the Amber ff99SB force field of Chimera [41]. At this point, the structure was considered ready for protein–ligand docking.

### 4.2. Pharmacophore Generation

Furthermore, the ligands were aligned using the alignment perspective tool in LigandScout (v. 4.4), their key structural features were merged, and various pharmacophore models were generated based on their multi-conformer [42]. To do so, the FAST settings and the Pharmacophore RDF-Code Similarity algorithms were applied to obtain all the hypotheses [42]. Additionally, the pharmacophore fit, the number of omitted structures, and the atom overlaps were set to 4, limiting only the best scoring models for further studies and rejecting the remaining worst scored models [43]. 

### 4.3. Virtual Screening and ADMET Virtual Filtration

Next, the obtained models were submitted for screening as a 3D query in the ZINCPharmer search database (http://zincpharmer.csb.pitt.edu/, accessed on 14 September 2021), specifically the ZINC purchasable subsection from a training set with over 21,777,093 compounds [44]. This software provided the results based on the predicted binding affinity of the database ligands against the protein (PDB#6I3C) using docking algorithms. To filter the selection, only the ligands with all the desired structural moieties shared with the training set were considered hit molecules. Moreover, to enhance the possibilities of discovering potential new drug-like leads, the results were also subjected to a Lipinski’s Rule of Five properties filter to improve the results obtained [45,46], analyzing the compounds’ molecular weight, their logP, and the number of rotatable bonds and hydrogen bond acceptor/donor groups. From there, 100 hit molecules were selected to be further analyzed by molecular docking and to study their predicted ADMET properties to screen for potential false positive.

### 4.4. Molecular Docking

To further improve the reliability of the model, a redocking of the top 100 hit molecules was performed using the AutoDock Tools 4 software (v. 4.2.6) [47]. This study allowed for the screening of potential false positive hits, as well as to better understand the ligands’ interactions with the COMT active site. The 3D grid box (x = 40; y = 40; z = 40) was optimized to fit all the study hits using a grid spacing of 0.375 Å and was centered in the protein active site, identified by the binding site of the inhibitor 3,5-DNC. The binding poses were generated using the Lamarckian genetic computational algorithm. The hits were ranked based on their binding energies and their binding free energy (ΔG), expressed in kcal/mol by the software, and their atomic interactions were further studied using the PMV (v. 1.5.6) tool and the Discovery Studios (v. 4.5) program to better visualize and identify the most important atomic interactions [29].

### 4.5. ADMET Property Analysis

The ADMET properties were predicted using the webserver pkCSM (http://biosig.unimelb.edu.au/pkcsm/prediction, accessed on 3 October 2021) [48]. This server compares the data from approved FDA drugs and experimental compounds to predict the compounds’ main pharmacokinetic features. These include the BBB and CNS permeability, the drug’s potential human intestinal absorption, whether it is a P-glycoprotein substrate/inhibitor, and its hepatoxicity, among other properties used to filter potentially toxic drugs.

### 4.6. Materials, Reagents, and Solutions for In-Vitro Studies

Ultrapure reagent-grade water was obtained with a Milli-Q system (Milipore/Waters). Yeast nitrogen base (YNB), glucose, agar, yeast extract, peptone, glycerol, dithiotreitol (DTT), protease inhibitor cocktail, *S*-adenosyl-_L_-methionine (SAM), epinephrine (bitartrate salt), *DL*-methanephrine hydrochloride, citric acid monohydrate, and glass beads (500 µm) were purchased from Sigma Chemical Co. (St. Louis, MO, USA). The tested compounds ZINC302226, ZINC98288, ZINC825166420, ZINC27985035, and ZINC78496496 were purchased from MolPort (Riga, Latvia). All chemicals used were of analytical grade, commercially available, and used without further purification.

The five selected tested compounds (Table 5) to be studied were dissolved in DMSO in a final concentration of 10 mM and stored at 4 °C with protection from light. From this solution, several dilutions were prepared in order to obtain the final desired concentrations to be tested. The final DMSO concentrations used were 1% (cytotoxic assays) and 5% (enzymatic assays), concentrations with no significant effect on cytotoxicity studies and enzyme bioactivity, as previously tested (data not shown). 

### 4.7. In-Vitro MBCOMT Inhibition Assays

#### 4.7.1. Biosynthesis and Recuperation of MBCOMT

The production of the human recombinant MBCOMT was performed according to the procedure described by Pedro et al. [49]. Briefly, transformed *Komagataella pastoris* X33 with the expression vector was grown for 72 h at 30 °C in yeast extract peptone dextrose (YPD) medium plates with 200 µg/mL of Zeocin. A single colony was inoculated in 100 mL of buffered minimal glycerol medium (BMGH) in 500 mL shake flasks. Cells were grown at 30 °C and 250 rpm to a cell density of 600 nm (OD_600_) of 6.0 units. Afterwards, an aliquot was introduced into 100 mL of buffered minimal methanol medium (BMMH) in 500 mL shake flasks, with an initial OD_600_ fixed to 1.0 unit. After 24 h of growth at 30 °C and 250 rpm, cells were collected by centrifugation (1500× *g*, 10 min, 4 °C). The cells were resuspended in lysis buffer (150 mmol L^−1^ NaCl, 10 mmol L^−1^ DTT, 50 mmol L^−1^ Tris, 1 mmol L^−1^ MgCl_2_, pH 8.0) supplemented with protease inhibitor cocktail. Then, a mechanical treatment with glass beads (7 cycles of vortexing for 1 min with 1 min of interval on ice) was applied to disrupt the cells. Cell suspensions were centrifuged (500× *g*, 5 min, 4 °C). The supernatant was removed, and the pellet obtained was resuspended in the same lysis buffer, without DTT. The samples were stored at 4 °C until use for further assays.

#### 4.7.2. MBCOMT Enzymatic Assay

The methylation efficiency of MBCOMT, alone or in the presence of the tested compounds, was evaluated by the amount of metanephrine converted from the substrate epinephrine, as described by Vieira-Coelho and Soares-da-Silva, with minor alterations [33]. Briefly, MBCOMT lysates with a fixed concentration of 1 mg/mL were preincubated at 37 °C for 20 min with increasing concentrations of the selected tested compounds (Table 5) before the addition of the substrate epinephrine (after 15 min) in the presence of SAM, EGTA, and sodium phosphate buffer (pH 7.8). The reaction was then stopped with 2 M of perchloric acid. After 1 h at 4 °C, samples were centrifuged (6000 rpm, 10 min, 4 °C) and the supernatant was filtered through a 0.22 µm cellulose acetate pore filter. The metanephrine levels in the sample were determined by HPLC with coulometric detection, as previously described [50]. All data analysis was performed using Prism 6 (GraphPad Software Inc. San Diego, CA, USA).

### 4.8. Cytotoxicity Studies

#### 4.8.1. Cell Cultures

The cell lines used were normal human dermal fibroblasts (NHDF) and an N27 rat dopaminergic neural cell line (N27), both obtained from American Type Culture Collection (ATCC). The cultures were performed in 75 cm^3^ culture flasks in an incubator at 37 °C under a humidified atmosphere with 5% carbon dioxide. The NHDF cell line was cultured in Roswell Park Memorial Institute (RPMI) 1640 culture medium (Sigma Aldrich, St. Louis, MO, USA) supplemented with 10% fetal bovine serum (FBS), 2 mM of *L*-glutamine, 10 mM of HEPES, 1 mM of sodium pyruvate, and 1% antibiotic Ab (Sigma Aldrich; 10,000 U/mL of penicillin G, 100 mg/mL of streptomycin, and 25 µg/mL of amphotericin B). The N27 cell line was cultured in RPMI 1640 culture medium with 10% FBS, 2 mM of *L*-glutamine, and 1% antibiotic Sp (Sigma Aldrich; 10,000 U/mL of penicillin G, 100 mg/mL of streptomycin). The medium was renewed every 2–3 days for NHDF and every day for N27 until cell confluence was 90–95%. Then, cells were incubated for 2–3 min with a solution of trypsin (0.5 g/L trypsin with 0.02 g/L of ethylene diamine tetra-acetic acid (EDTA)) to detach cells from the bottom of the culture flask. Afterwards, the suspension of cells in trypsin solution was diluted with an appropriate medium and centrifuged for 5 min at 125 G. Then, cells were counted by Trypan-blue exclusion method using a Neubauer chamber, resuspended, and seeded at 2 × 10^4^ cells/mL density in 96-well culture plates (VWR) with a volume of 100 µL per well. The cells were kept in culture for a period of 48 h for cell adherence and growth before cytotoxicity studies.

#### 4.8.2. MTT Assay

The cytotoxicity was assessed by the 3-(4,5-dimethylthiazol-2-yl)-2,5-diphenyltetrazolium bromide (MTT) colorimetric assay, performed as previously described [51]. For this, after adherence and growth, cells were exposed during 48 h to the compounds under study as well as 5-fluorouracil (5-FU; positive control) at different concentrations (0.1, 1, 10, 30, 50, and 100 µM) for dose-response studies. These solutions were prepared from stock solutions at 10 mM in DMSO by dilution in the appropriate cell culture medium. Then, the experimental medium was replaced with 100 µL per well of an MTT solution (1 mg/mL of MTT concentration, 20% phosphate buffer saline (PBS), and 80% culture medium without FBS and antibiotics). After 4 h of incubation, the MTT solution was replaced by DMSO to solubilize formazan crystals. The absorbance of each well was measured at 570 nm using a microplate spectrophotometer Bio-Rad xMark. The results are expressed as relative cell viability (%) normalized to negative control and considering standard deviation. At least two independent assays in quadruplicate were performed for each stimulus. Differences between treatments were analyzed with Student’s *t*-test and the results were considered statistically significant when the *p*-value < 0.05. Half maximal inhibitory concentrations (IC_50_) were determined through sigmoidal dose-response curve fit considering a 95% confidence interval.

## 5. Conclusions

PD is the second most common neurodegenerative disorder, and in most situations its cause is unknown. Its prevalence is between 100 and 300/100,000 inhabitants, and the number of PD patients is expected to double by 2030 [52]. The search for new drugs that improve the patient’s quality of life and prevent the progression of the disease is ceaseless on the part of the pharmaceutical industry. The advantage of the use of computational chemistry in comparison with experimental laboratory methods is that it reduces expenses derived from the purchase of materials as well as the production of polluting compounds, and allows the research process to be accelerated, for example, in the preclinical stages of drug development. In the present work, a set of molecules with potential interest in PD acting as COMT inhibitors was discovered through a computational-based approach. The five selected molecules for in-vitro studies revealed relevant and selective MBCOMT inhibitory effects. In addition, cytotoxicity evaluation in dopaminergic (N27) and fibroblast (NHDF) cells evidenced that selected hits have relatively low toxicity. Within this group, the best results were observed for the compound ZINC27985035, with an IC_50_ of 17.6 nM determined for MBCOMT inhibition and low cytotoxicity observed in both cell lines (61.26 and 40.31 μM, respectively). Despite this compound having been previously described as a COMT inhibitor, no studies have been performed on membrane COMT isoform. In addition, the other four selected compounds have not been previously studied against this target to our knowledge, which can open new medicinal chemistry possibilities in the design of new COMT inhibitors. The relevant results obtained, combined with the similarity in structure to the commercial COMT inhibitor entacapone, may allow the future development of potential new drug candidates for PD with improved properties.

## Figures and Tables

**Table 1:** Hit molecule predicted values of the binding affinities and main interactions with the target.

Compound	Binding Energy	Main Interactions
ZINC825166420	−7.48	Met40, Pro174, Glu199
ZINC1547205762	−7.39	Trp38, Met40, Pro174, Mg^2+^
ZINC98288	−6.98	Trp38, Met40, Lys144, Pro174, Glu199
ZINC95473053	−6.79	Trp38, Met40, Asp141, Trp143, Lys144, Asn170, Pro174, Glu199, Mg^2+^
ZINC302226	−6.63	Met40, Pro174, Glu199, Mg^2+^
ZINC27985035	−6.26	Met40, Asp141, Lys144, Asn170, Pro174,
ZINC78496496	−6.12	Trp38, Met40, Pro174, Leu198, Glu199, Mg^2+^
ZINC38175059	−6.04	Trp38, Met40, Asp141, Lys144, Asn170, Pro174, Leu198, Glu199
ZINC605706834	−6.24	Trp38, Trp143, Pro174
ZINC68675288	−5.90	Met40, Asn170, Pro174, Glu199

**Table 2:** ADMET prediction for the most promising compounds.

Compound	Intestinal Absorption (%)	PgP Substrate	PgP I/II Inhibitor	BBB Permeability (logBB)	CNS Permeability (log PS)	CYP2D6 Substrate	CYP3A4 Substrate	CYP1A2 Inhibitor	CYP2C19 Inhibitor	CYP2C9 Inhibitor	CYP2D6 Inhibitor	AMES Toxicity	LD50 (mol/kg)	LOAEL	Hepatoxicity
ZINC825166420	91.048	Yes	No/No	−0.013	−1.625	No	Yes	Yes	Yes	Yes	No	Yes	2.563	2.278	Yes
ZINC1547205762	84.913	Yes	No/No	−1.601	−3.777	No	No	Yes	Yes	Yes	Yes	No	2.346	2.323	No
ZINC98288	88.908	Yes	No/No	−0.722	−2.205	No	No	Yes	Yes	No	No	No	2.017	2.085	No
ZINC95473053	97.282	Yes	Yes/Yes	−1.467	−3.022	No	Yes	Yes	Yes	Yes	Yes	Yes	2.444	2.511	No
ZINC302226	69.066	Yes	No/No	−1.669	−2.427	No	No	Yes	No	Yes	No	No	2.67	3.036	No
ZINC27985035	76.88	Yes	No/No	−0.356	−2.587	No	No	No	No	No	No	Yes	2.044	2.407	No
ZINC78496496	77.717	Yes	No/No	−0.603	−2.601	No	No	No	Yes	No	No	Yes	2.154	2.329	No
ZINC38175059	71.419	Yes	No/No	−0.569	−2.78	No	No	No	No	No	No	Yes	1.804	2.158	No
ZINC605706834	63.604	Yes	No/No	−0.813	−2.724	No	No	No	No	No	No	No	2.655	2.538	No
ZINC68675288	53.332	Yes	No/No	−1.278	−4.074	No	No	No	No	No	No	No	2.474	2.537	No

**Table 3:** Estimated IC_50_ values (nM) for selected compounds with MBCOMT performed in this work in comparison with previous ones reported in the literature. Values are means with 95% confidence (*n* = 2 to 3).

Sample	Compound	IC50 (nM)	Reference
Recombinant SCOMT	3,5-DNC	13.26 (10.7 to 16.44)	[32]
	Entacapone	4.224 (2.949 to 6.050)	
Brain MBCOMT	Tolcapone	2 (1 to 2)	[33]
Liver MBCOMT	Tolcapone	123 (52 to 292)	
Liver SCOMT	Pyrazoline derivate	48	[34]
	Entacapone	230	
Recombinant MBCOMT	ZINC302226	1083 (879.9 to 1333)	This work
	ZINC98288	943.8 (816 to 1092)	
	ZINC825166420	1538 (1254 to 1888)	
	ZINC27985035	17.6 (13.53 to 22.96)	
	ZINC78496496	470 (401.4 to 550.4)	

**Table 4:** Estimated IC_50_ values (µM) for selected compounds in dopaminergic neuronal (N27) and normal fibroblast (NHDF) cells ^a^.

Compound	N27	NHDF
ZINC302226	52.50	>100
ZINC98288	69.78	>100
ZINC825166420	16.98	12.14
ZINC27985035	61.26	40.31
ZINC78496496	>100	92.90
5-FU	4.28	5.16

^a^ Cells were treated with different concentrations (0.1, 1, 10, 30, 50, and 100 µM) during 48 h. The cell proliferation effects were determined by the MTT assay. The data shown are representative of at least two independent experiments. 5-FU: 5-fluorouracil.

**Table 5:** Compounds and concentrations to be used in the in-vitro studies of enzymatic activity and cytotoxicity.

Compound	Concentrations for Cytotoxicity Assays (µM)	Concentrations for Bioactivity Assays (µM)
ZINC302226	0.1 to 100	0.125 to 10
ZINC98288		0.0315 to 10
ZINC 825166420		0.125 to 20
ZINC27985035		0.0078 to 0.5
ZINC78496496		0.125 to 10

## Data Availability

Data is contained within the article.

## Supplementary Materials

The following supporting information can be downloaded at: https://www.mdpi.com/article/10.3390/ph15010051/s1. Table S1. Characteristics of the studied compounds; Table S2. Virtual screening results of the hit molecules obtained in ZINCPharmer; Table S3. Results of in vitro MBCOMT inhibition evaluation of the 10 best scored selected compounds by in silico studies; Figure S1. Graphics of concentration-response studies in dopaminergic N27 cell line; Figure S2. Graphics of concentration-response studies in normal human dermal fibroblasts (NHDF).
