# Supplementary material for: Discovery of Small Molecules as Membrane-Bound Catechol-O-methyltransferase Inhibitors with Interest in Parkinson’s Disease: Pharmacophore Modeling, Molecular Docking and In Vitro Experimental Validation Studies
Source: Pharmaceuticals (Basel). 2021 Dec 31;15(1):51. doi: 10.3390/ph15010051 (PMC8780549; doi:10.3390/ph15010051)

pharmaceuticals

Discovery of Small Molecules as Membrane-Bound Catechol-*O*-methyltransferase Inhibitors with Interest in Parkinson's Disease: Pharmacophore Modeling, Molecular Docking and In Vitro Experimental Validation Studies

Pedro Cruz-Vicente [1,2,†], Ana M. Gonçalves [1,2,3,†], Octávio Ferreira [1], João A. Queiroz [1], Samuel Silvestre [1,4,5,*], Luís A. Passarinha [1,2,3,5,*] and Eugenia Gallardo [1,5,*]

[1] CICS-UBI, Health Sciences Research Centre, Universidade da Beira Interior, 6201-001 Covilha, Portugal; pedromvcruz@hotmail.com (P.C.-V.); ggmargarida@gmail.com (A.M.G.); octavioferreira.2@gmail.com (O.F.); jqueiroz@ubi.pt (J.A.Q.)
[2] UCIBIO—Applied Molecular Biosciences Unit, Departamento de Química, Faculdade de Ciências e Tecnologia, Universidade NOVA de Lisboa, 2829-516 Caparica, Portugal
[3] Associate Laboratory i4HB—Institute for Health and Bioeconomy, NOVA School of Science and Technology, Universidade NOVA, 2819-516 Caparica, Portugal
[4] CNC—Center for Neuroscience and Cell Biology, University of Coimbra, 3004-504 Coimbra, Portugal
[5] Laboratório de Fármaco-Toxicologia—UBIMedical, Universidade da Beira Interior, 6200-001 Covilha, Portugal
* Correspondence: sms@ubi.pt (S.S.); lpassarinha@fcsaude.ubi.pt (L.A.P.); egallardo@fcsaude.ubi.pt (E.G.); Tel.: +351-275-329-002 (S.S. & L.A.P. & E.G.)
† These authors contributed equally to this paper.

Supplementary material

Table S1. Characteristics of the studied compounds.

Compound	RMSD (Å)	Molecular Weight (Da)	Routable bonds
ZINC92701879	1.049	298	7
ZINC302226	0.987	298	3
ZINC06520424	1.113	250	6
ZINC03022616	1.062	466	9
ZINC605706834	1.018	480	2
ZINC98288	0.890	257	3
ZINC07056138	1.074	302	6
ZINC68675288	0.755	236	1
ZINC63699107	0.950	323	3
ZINC825166420	1.150	277	2
ZINC72195935	0.662	293	7
ZINC78496496	0.977	262	2
ZINC00002021	0.951	348	7
ZINC92121085	0.960	336	6
ZINC09287838	0.975	307	3
ZINC38175059	0.669	213	2
ZINC95473053	0.879	473	5
ZINC72200820	0.782	213	9
ZINC94057102	0.897	327	6
ZINC38549642	0.730	339	7
ZINC16310105	0.953	445	6
ZINC27985035	0.697	180	1
ZINC1547205762	1.265	406	4
ZINC90891275	0.785	356	9
ZINC83307569	0.650	245	5
ZINC59246393	0.870	263	4

Table S2. Virtual screening results of the hit molecules obtained in ZINCPharmer.

Compound.	Binding energy (kcal/mol)	Intermolecular energy (kcal/mol)
ZINC4706996	-7.65	-9.14
ZINC4776068	-7.61	-8.51
ZINC825166420	-7.48	-8.67
ZINC1547205762	-7.39	-9.47
ZINC61887498	-6.98	-7.87
ZINC98288	-6.98	-8.77
ZINC301795	-6.88	-8.67
ZINC95473053	-6.79	-9.48
ZINC95477008	-6.78	-8.87
ZINC68677292	-6.78	-7.68
ZINC38515687	-6.74	-8.23
ZINC4783172	-6.7	-7.6
ZINC302226	-6.63	-8.12
ZINC4522753	-6.58	-7.48
ZINC72194201	-6.52	-7.72
ZINC5123812	-6.52	-8.31
ZINC4770553	-6.48	-7.67
ZINC5699358	-6.48	-7.67
ZINC27985035	-6.26	-7.15
ZINC605706834	-6.24	-7.05
ZINC71404891	-6.15	-7.04
ZINC78496496	-6.12	-7.32
ZINC95836232	-6.07	-8.76
ZINC1611750	-6.06	-7.25
ZINC38175059	-6.04	-7.53
ZINC72194198	-6.01	-7.2
ZINC68675288	-5.9	-7.1
ZINC78496489	5.85	-7.34
ZINC478514	-5.82	-7.9
ZINC72194202	-5.67	-7.17
ZINC2038230	-5.37	-6.56
ZINC95635255	-5.2	-6.69
ZINC27977563	-5.11	-6.6
ZINC34064982	-5.06	-6.55
ZINC90685655	-4.96	-6.16
ZINC82401665	-4.94	-6.13
3,5-DNC	-4.85	-6.05
ZINC90598152	-4.63	-6.12
ZINC95836233	-4.53	-6.32
ZINC337530	-4.27	-6.06
ZINC3861001	-3.89	-7.17

Table S3. Results of *in vitro* MBCOMT inhibition evaluation of the 10 best scored selected. compounds by *in silico* studies.

Tested compounds	Concentration (μM)	Inhibition (%)
ZINC302226	10	56
	100	100
ZINC605706834	10	39
	100	34
ZINC98288	10	100
	100	100
ZINC68675288	10	40
	100	43
ZINC825166420	10	72
	100	100
ZINC1547205762	10	14
	100	27
ZINC95473053	10	41
	100	46
ZINC27985035	10	100
	100	100
ZINC78496496	10	81
	100	100
ZINC38175059*	10	100
	100	100

*This compound has not advanced for IC_{50} determinations due to the lower affinity observed in docking studies.

Figure S1. Graphics of concentration-response studies in dopaminergic N27 cell line.

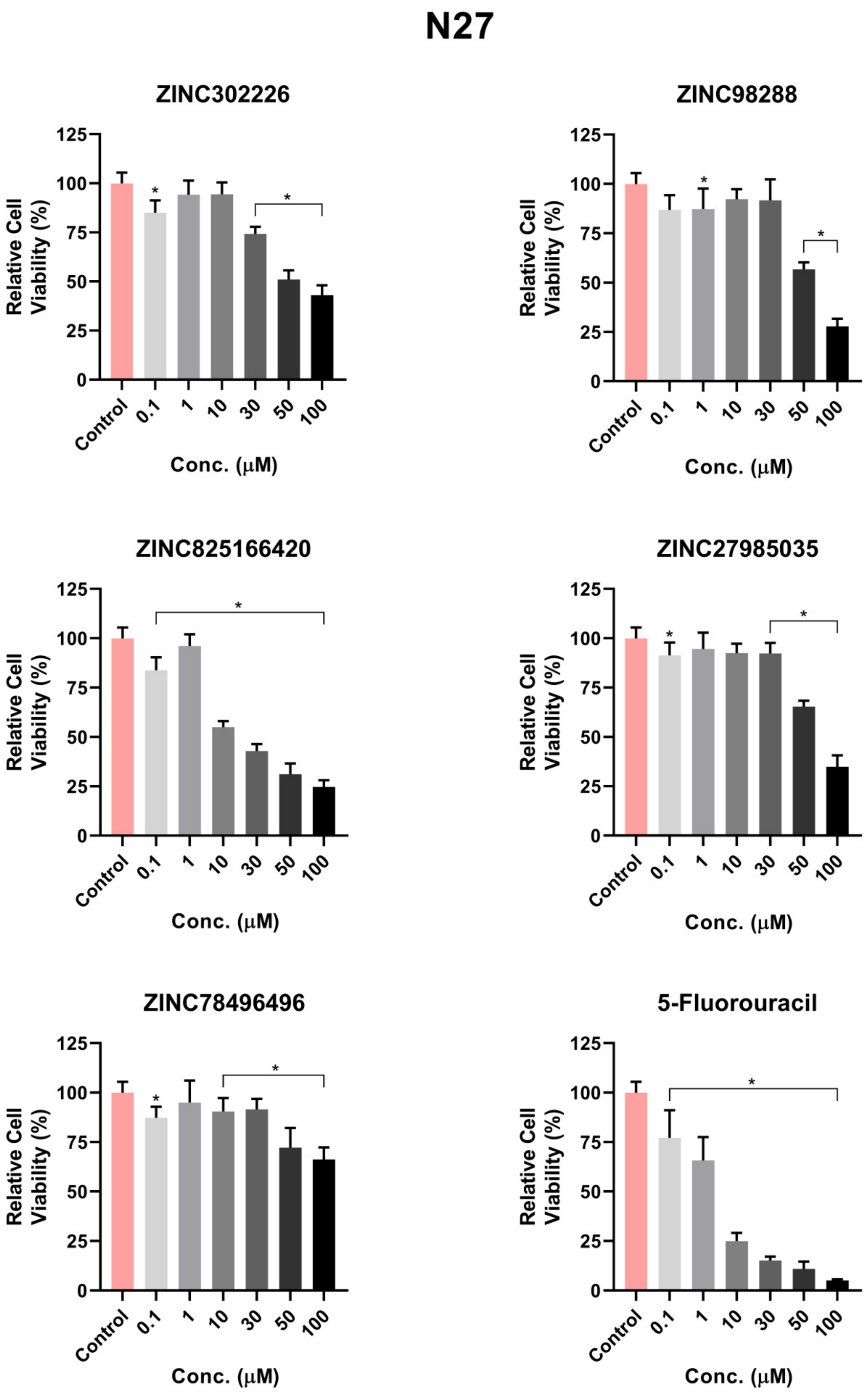

Figure S2. Graphics of concentration-response studies in normal human dermal fibroblasts (NHDF).

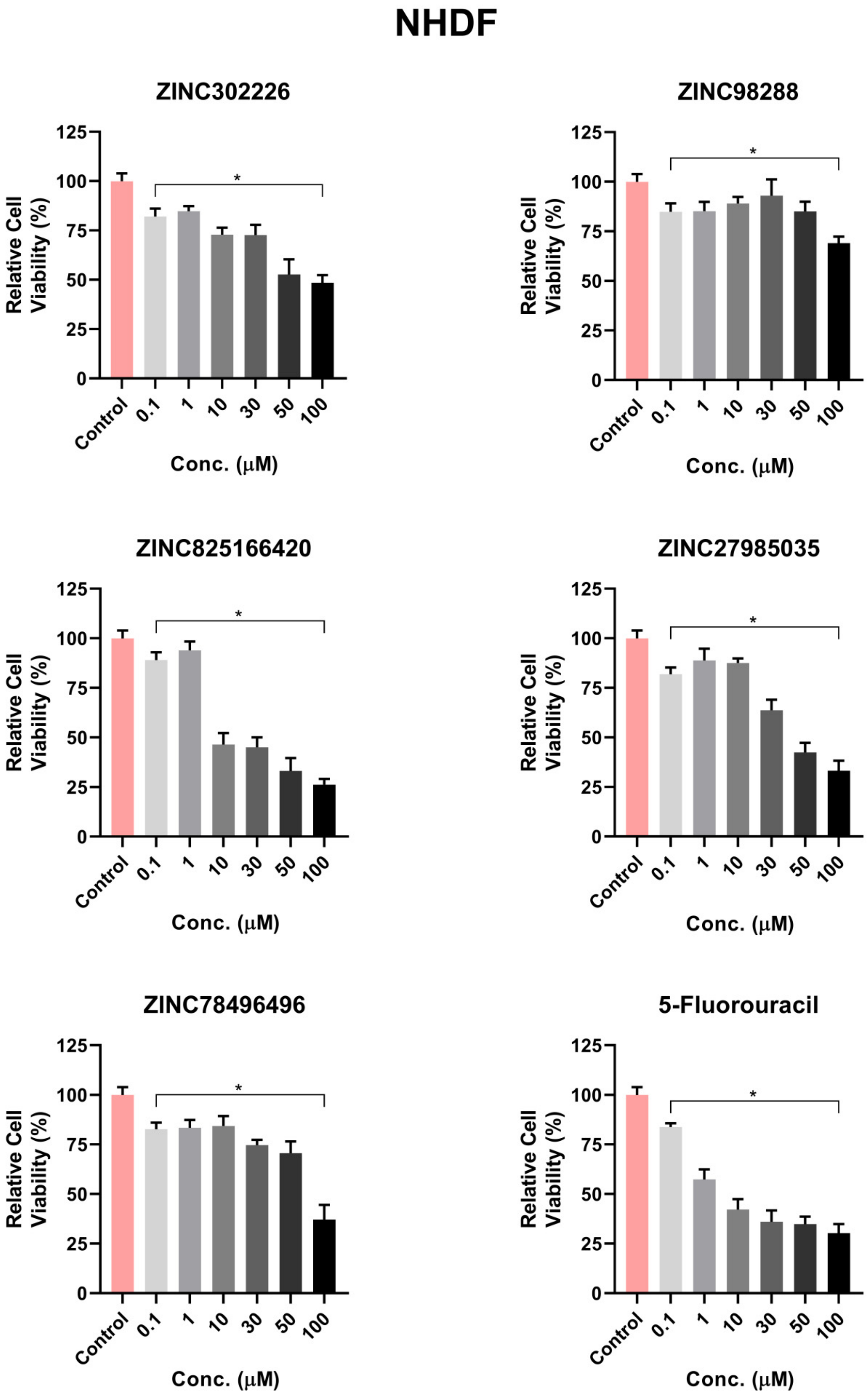

Supplement: Supplementary file 1 [file pharmaceuticals-15-00051-s001.zip › pharmaceuticals-1512241-supplementary.pdf]
